# Light-Activated Iron Oxide Nanoparticles in Cancer Treatment: Synergistic Roles in Photothermal and Photodynamic Therapy

**DOI:** 10.3390/cancers18081203

**Published:** 2026-04-09

**Authors:** Aynura Karimova, Habiba Shirinova, Toghrul Sadikhov, Javahir Hajibabazade, Sabina Hajizada, Yerkeblan Tazhbayev, Abdumutolib A. Atakhanov, Samir N. Babayev, Christoph Reissfelder, Vugar Yagublu

**Affiliations:** 1 Department of Chemistry of High Molecular Weight Compounds, Baku State University, Baku AZ 1148, Azerbaijan; aynurakarimova@bsu.edu.az; 2 Department of Chemical Physics of the Nanomaterials, Baku State University, Baku AZ 1148, Azerbaijan; habiba.shirinova@bsu.edu.az; 3 Department of Molecular Biology and Genetics, Institute of Graduate Studies in Sciences, Istanbul University, 34134 Istanbul, Türkiye; toghrul.sadikhov@ogr.iu.edu.tr; 4 Department of Food Safety and Risk Assessment, Scientific Research Centre, Azerbaijan Food Safety Institute, Baku AZ 1124, Azerbaijan; 5 Carver College of Medicine, University of Iowa, Bowen Science Building, 51 Newton Road, Iowa City, IA 52242, USA; javahir-hajibabazade@uiowa.edu; 6 Department of Surgery, Universitätsmedizin Mannheim, Medical Faculty Mannheim, University of Heidelberg, 68167 Mannheim, Germany; sabina.hajizada@medma.uni-heidelberg.de (S.H.); christoph.reissfelder@umm.de (C.R.); 7 Scientific Research Centre, Azerbaijan Medical University, Baku AZ 1022, Azerbaijan; 8 Institute of Chemical Problems, Karagandy University of the Name of Academician E.A. Buketov, Karaganda City 100028, Kazakhstan; tazhbayev.y@karnu-buketov.edu.kz; 9 Institute of Polymer Chemistry and Physics, Uzbekistan Academy of Science, Tashkent 100128, Uzbekistan; a-atakhanov@academy.uz; 10 Department of Obstetrics and Gynecology, Karabakh University Clinic, Khankendi AZ 2600, Azerbaijan; samir.babayev@karabakh.edu.az; 11 Faculty of Medicine and Health Sciences, Karabakh University Clinic, Khankendi AZ 2600, Azerbaijan

**Keywords:** Fe_3_O_4_ nanoparticles, light-based cancer therapy, photothermal therapy, photodynamic therapy, ROS, Fenton-like reactions, photosensitizer delivery

## Abstract

Iron oxide nanoparticles have emerged as multifunctional compounds with prominent potential in cancer theranostics, particularly in photothermal therapy (PTT) and photodynamic therapy (PDT). Their unique electronic and crystal structures, such as the dispersion of Fe^2+^ and Fe^3+^ ions and d-orbital splitting, contribute to their magnetic and catalytic properties. In PTT, Fe_3_O_4_ nanoparticles exhibit moderate near-infrared (NIR) absorption and photothermal conversion efficiency, which can be enhanced through adjustments in particle size, surface modification, and combinations with other components. In PDT, Fe_3_O_4_ nanoparticles demonstrate intrinsic peroxidase-like catalytic activity, facilitating Fenton and photo-Fenton reactions that generate reactive oxygen species (ROS), including hydroxyl radicals (^•^OH), thereby amplifying oxidative stress in cancer cells. These nanoparticles can also function as carriers for photosensitisers (PS), promoting targeted delivery and enhanced ROS generation. Multifunctional nanomaterials that integrate Fe_3_O_4_ with other therapeutic agents and targeting ligands have demonstrated synergistic antitumour effects through amplified photothermal, photodynamic, chemodynamic, and chemotherapeutic mechanisms. Despite certain drawbacks, such as relatively low NIR absorption and challenges in optimising delivery and light activation, ongoing improvements in Fe_3_O_4_-based nanoplatforms present significant potential for enhancing treatment outcomes and the precision of cancer therapy. This article systematically explores the synergistic role of Fe_3_O_4_ nanoparticles in PTT and PDT, encompassing their magnetic and catalytic characteristics. Additionally, it focuses on multifunctional hybrid nanoplatforms that combine Fe_3_O_4_ with targeting or imaging agents, highlighting their potential to enhance therapeutic precision.

## Simple Summary

Treating cancer effectively without harming healthy tissues remains a major challenge in modern medicine. Light-based therapies provide a more focused approach, but their effectiveness is often limited when applied individually. In this review, we describe how iron oxide nanoparticles can help overcome these limitations by bringing several therapeutic functions together in one system. Under light exposure, these nanoparticles can generate heat and reactive oxygen species that damage cancer cells, while also carrying therapeutic molecules. Their magnetic properties further allow them to be guided toward tumour sites, improving targeting accuracy. By combining these capabilities, such systems offer a promising way to enhance treatment efficiency and reduce unwanted side effects. This work highlights their potential role in the development of more precise and effective cancer therapies.

## 1. Introduction

Cancer is a leading cause of mortality worldwide. In 2022, there were 9.7 million cancer-related deaths, and the World Health Organisation (WHO) estimates the annual incidence to exceed 35 million by 2050 [1]. Cancer progression is complex and characterised by the progressive accumulation of genetic and epigenetic alterations in cells, resulting in uncontrolled cell division, growth, invasion, and metastasis. For many years, cancer treatment options have included surgery, radiation therapy, chemotherapy, and immunotherapy, used individually or in combination [2]. While surgery remains a primary treatment for localised solid tumours, many cancers metastasise, necessitating more intensive systemic therapies such as chemotherapy and radiotherapy. These conventional therapies typically require high dosages of drugs and radiation, which are detrimental to the healthy tissues surrounding tumours and lead to adverse systemic side effects.

To mitigate the treatment-related burdens, more precise and less invasive treatment methods need to be employed. In this context, advanced nanotechnology and targeted therapies have been developed as promising approaches, utilising different types of nanoparticles (NPs) with diverse physicochemical properties. These NPs include metal, semiconductor, carbon, and organic NPs, with sizes ranging from a few to hundreds of nanometres [3,4,5,6]. Improved knowledge of the NP’s application properties has led to further advancements in light-based cancer treatments, such as PTT and PDT [7,8,9]. These perspectives enable selective activation of therapeutic photothermal agents (PTA) at the tumour site in a tumour-specific manner, reducing systemic toxicity and increasing therapeutic efficacy. For instance, the combination of iron oxide NPs with light-based therapies offers a dual benefit: improved targeted therapy and minimally invasive treatment, making cancer therapies safer and more effective [10,11]. The unique properties of iron oxide NPs, such as high surface energy, biocompatibility, stable sorption of biomolecules, the capability to alter physicochemical properties under the influence of physical fields, and the presence of magnetic properties, enhance their therapeutic potential [12,13,14,15]. For instance, high surface energy enables NPs to stably adsorb therapeutic molecules, including photosensitisers or drugs, onto their surfaces [16]. This makes them effective carriers in PDT, where they can deliver ROS generators to cancer cells upon light activation, thereby enhancing localised treatment efficacy. Further, the biocompatibility of iron oxide NPs ensures safe interaction with biological systems, enabling repeated or prolonged therapeutic use, which is critical in light-based therapies [17,18]. These NPs also exhibit changes in physicochemical properties when exposed to, for example, magnetic fields, enhancing their ability to convert light into localised heat in PTT [11].

In recent years, a substantial number of review and research articles have been published in the fields of PTT and PDT. However, an analysis of studies conducted over the last two decades indicates limited integrated and systematic investigation of these two light-based therapeutic modalities within a unified therapeutic framework. The majority of existing reviews discuss PTT and PDT separately or evaluate the potential of NPs for each therapy individually. Conversely, a comparative analysis of the synergistic mechanisms of PTT and PDT within a single system, considering the physical fundamentals of light–NP interactions, light parameters, and the structural characteristics of the nanoplatform, has not been sufficiently addressed in the literature. Particularly, comprehensive reviews that systematically examine the use of Fe_3_O_4_ NPs as multifunctional platforms for both PTT and PDT, alongside the advantages and limitations of this approach, are scarce. This review article aims to bridge this gap by providing a mechanism-oriented and comparative overview of the potential of Fe_3_O_4_-based nanoplatforms for PTT and PDT applications.

This review explores the integration of Fe_3_O_4_ NPs with light-based cancer therapies, highlighting their synergistic effects in improving treatment efficacy. It addresses the essential principles of light absorption and tissue penetration relevant to PTT and PDT, clarifying the core mechanisms of heat and ROS generation. Here, we analyse the role of Fe_3_O_4_ NPs in PTT and PDT, encompassing their electronic structure, magnetic, and catalytic characteristics. Multifunctional hybrid nanoplatforms combining Fe_3_O_4_ with targeting or imaging agents are also discussed regarding their potential to enhance therapeutic precision.

## 2. Light Absorption and Tissue Penetration

The intensity of the thermal effect depends on the amount of absorbed light energy and the depth of tissue penetration, both of which are influenced by the laser wavelength. Laser sources with wavelengths in the red and NIR regions are typically employed, as this spectral range enables sufficiently deep penetration into biological tissues. In this spectral region (650–950 nm and 1000–1350 nm), key cellular components, such as haemoglobin, water, lipids, and melanin, exhibit low absorption and scattering coefficients, enabling efficient light propagation through biological tissues [19,20,21].

The Beer–Lambert Law (BLL) describes the relationship between light absorption in biological tissues. It explains how the intensity of light diminishes as it passes through an absorbing medium, which is critical for understanding and optimising laser-based therapies. It is expressed as:

(1) $$I=I_{0}\cdot exp\left(-\mu_{a}d\right)$$

where

*I*_0_ is the intensity of the incident light;

*I* is the intensity of the transmitted light;

*μ_a_* is the absorption coefficient (cm^−1^) of the medium;

*d* is the thickness of the medium through which the light passes [22].

Extensions and modifications of the BLL, considering various factors affecting light–tissue interactions, were discussed by Oshina and Spigulis [23]. Considering the reflectance at the air–tissue interface *R*, the *μ_a_* can be expressed through the following equation:

(2) $$\mu_{a}=-\frac{1}{d}\left[ln\left(\frac{I}{I_{0}}\right)-ln\left.{\left(\left(1-R^{2}\right.\right)}^{2}\right)\right]$$

The absorption coefficient is also called the tissue attenuation coefficient, and its inverse value represents the depth of light penetration into the skin. Penetration depth is a measure of how deep light or any electromagnetic radiation can penetrate the skin [24]. When electromagnetic radiation strikes the skin’s surface, it may be partially reflected, with the remaining energy transmitted into the tissue as an energy field. Generally, penetration depth varies with wavelength. Moreover, repeated backscattering and total internal reflection at the tissue–surrounding medium interface can significantly alter the actual light penetration depth. Notably, the values of the absorption coefficient and penetration depth may generally vary depending on the optical and physical properties of the tissue under investigation.

Bashkatov et al. [25] calculated the penetration depth *δ* through the following equation:

(3) $$\delta =\frac{1}{\sqrt{3\mu_{a}\left(\mu_{a}+\mu_{s}^{\prime }\right)}}$$

where $\mu_{s}^{\prime }$ is the scattering coefficient.

Douplik et al. [26] show that a wavelength of 1090 nm yielded the maximum penetration depth of 3.5 mm. At wavelengths of 600, 633, 660, 700, 750, 800, 850, and 900 nm, the penetration depths were reported as 1.5, 1.7, 1.8, 2.0, 2.2, 2.3, 2.4, and 2.5 mm, respectively. As previously discussed, longer wavelengths within the 600–1200 nm range fall within the optical transparency window of biological tissues, enabling greater penetration depths [27].

These observations confirm the strong dependence of light penetration depth on wavelength, emphasising the importance of selecting laser parameters within the tissue optical window. Accurate modelling of light absorption and scattering is, therefore, essential for optimising energy delivery in light-based therapeutic applications.

## 3. Photothermal Therapy (PTT)

PTT involves the thermal destruction of targeted tissue using a focused beam of electromagnetic radiation emitted by a laser [28]. It is closely related to other laser-based methods, such as laser interstitial thermal therapy (LITT), interstitial laser photocoagulation (ILP), and laser ablation (LA). While these terms are sometimes used interchangeably due to their shared mechanism of applying laser-induced heat to destroy tissue, each method has unique technical specifications and clinical applications [29,30,31,32,33,34].

The PTT technique relies on the photothermal effect, which occurs when cells absorb light energy, causing a sharp increase in temperature in the treated area. The heat generated by various mechanisms, such as plasmon resonance and energy transfer, selectively raises the local temperature to levels that cause irreversible cellular damage, ultimately leading to tissue necrosis [35,36].

**Figure 1:**
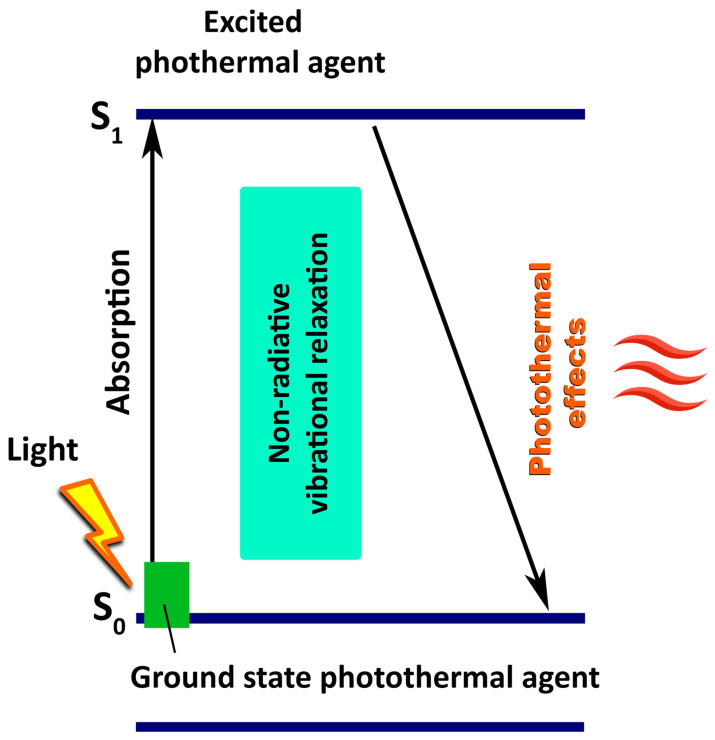
Schematic representation of light-induced non-radiative relaxation and heat generation in PTT.

PTT’s fundamental mechanism begins with the absorption of light by PTAs, particularly in the NIR region, followed by a sequence of molecular relaxation events ultimately resulting in heat generation. When light of an appropriate wavelength irradiates PTAs, it is excited from the singlet ground state (S_0_) to a higher electronic singlet excited state (S_1_, S_2_, etc.) (Figure 1).

The excited state is typically unstable and has a limited lifetime, often in the range of nanoseconds. There may be different mechanisms (or pathways) of relaxation from the excited state to the ground state, depending on the type of PTAs, the energy of the absorbed photon, and the nature of the environment. According to the Jablonski diagram, relaxation from the excited state may proceed through radiative and non-radiative processes [37]. PTT relies predominantly on non-radiative relaxation mechanisms, as they efficiently convert absorbed photon energy into heat, which is essential for inducing localised thermal damage in target tissues [38,39,40,41].

Upon photon absorption, molecular vibrations are induced, leading to the generation of heat. Subsequently, this vibrational energy is transferred to the surrounding medium, resulting in localised temperature elevation. In most cases, the energy is ultimately converted into thermal energy. Photothermal conversion efficiency (PCE) *η* is a critical parameter for evaluating the transformation of absorbed light energy into released thermal energy:

(4) $$\eta =\frac{Q}{E}=\frac{c_{p}mT}{pst}$$

where

*Q* is the thermal energy generated by the PTA;

*E* is the total incoming light energy;

*c_p_* is the specific heat capacity of the PTA;

*m* is the mass of the PTA;

Δ*T* is the observed temperature rise during

Irradiation;

*p* is the light source’s power density;

*s* is the irradiation area;

*t* is the duration [42,43].

Paściak et al. [44] analysed and compared different models for describing light-to-heat PCE. Their study revealed that PCE depends on numerous measurement conditions and assumptions, such as the system mass and geometry, the presence of colloidal sample stirring, and the method and location of temperature measurement.

Depending on the applied light intensity, PTT can generate different thermal regimes: ~55–100 °C (ablative/coagulative) or ~43–55 °C (sub-ablative), both capable of causing irreversible tissue injury [45,46]. Conversely, mild hyperthermia (~41–43 °C) is generally not tumouricidal per se but enhances the effectiveness of other treatments. It chemosensitises and radiosensitises tumours when applied for extended periods [47,48].

In clinical settings, accurate energy delivery and precise placement of laser fibres are vital to ensure effective tissue ablation while minimising damage to surrounding structures. In this context, Cepek and co-authors [49] evaluated the impact of laser fibre placement errors on the efficacy of therapy in achieving the desired volume of tissue destruction. The research used a simplified model of prostate cancer to simulate treatment conditions. They found that a target lesion with a length at least 5 mm shorter than the diameter of the tissue destruction zone can be fully ablated by using up to four laser fibres, provided the placement error does not exceed 3 mm. However, in this study, the location of the target lesion vis-a-vis the rest of the prostate, urethra, and rectum was not considered.

Considering the critical importance of accurate laser fibre placement for effective therapy, advanced imaging techniques play a key role in overcoming these challenges. Magnetic Resonance Imaging (MRI) has emerged as a powerful tool for performing PTT [50,51,52,53,54,55]. MRI guidance offers several advantages, including enhanced visualisation of the target area, precise navigation of the laser fibre, continuous monitoring, and control over the thermocoagulation zone and surrounding tissues [56]. Using real-time 3D MRI reconstructions in seven canine prostate models, Stafford and co-authors [57] demonstrated that the laser applicators could be positioned within 1.1 ± 0.7 mm of the target area. It is technically feasible to localise the laser fibre accurately within the target lesions, and real-time MRI during the destructive process enables a precise assessment of the extent of tissue necrosis [58].

Despite imaging advancements, a significant limitation of traditional hyperthermia and some PTT approaches is the lack of localisation. Conventional methods using external heat sources—such as thermal baths, microwaves, or radiofrequency waves—often generate temperature gradients that diminish with distance from the source. This lack of specificity can lead to unwanted thermal effects and damage to healthy tissue. Consequently, the use of exogenous or small-molecule PTAs has gained traction as a means of improving spatial precision in PTT [45,59].

An ideal PTA should possess several main characteristics that ensure its effectiveness and safety [60]. First, it should accumulate predominantly in tumour cells rather than being distributed throughout the body. Second, an ideal PTA should have a high PCE at the treatment wavelength. Furthermore, excellent biocompatibility is crucial for an optimal PTA. This includes minimal dark toxicity, meaning that the agent should not injure tissues outside the light-exposed region. Finally, the PTA should maintain its structural and functional integrity under repeated or prolonged exposure to light. This robust photostability might extend the applicability of the PTAs, allowing them to be used in multiple treatment sessions without additional administration.

### Nanomaterial-Based Photothermal Agents

Nanomaterials have proven to be highly effective in meeting these stringent requirements. They have particularly, been able to overcome the limitations of traditional PTT due to their ability to convert absorbed energy into heat efficiently. Moreover, this effect is particularly crucial in tumour vasculature, where leaky blood vessels facilitate the spatial accumulation of nanoparticles within the tumour tissue [61]. Therefore, these nanomaterials can destroy cancerous cells locally while minimising damage to healthy tissues.

**Figure 2:**
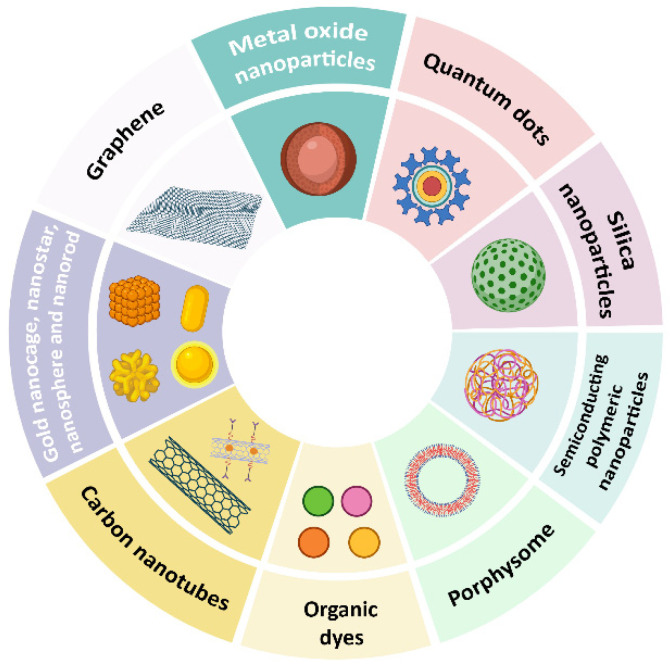
Different types of PTAs used for PTT. Created in BioRender. Sadikhov, T. (2025) https://BioRender.com/nk7rt97 (accessed on 15 October 2025).

Generally, nanomaterials as PTAs are classified into the following types (see Figure 2):

(i) Metal-based: gold (Au), silver (Ag), copper sulfide (CuS), and iron oxide (Fe_3_O_4_) nanoparticles [62,63,64];
(ii) Carbon-based: graphene, graphene oxide, and carbon nanotubes [65,66,67];
(iii) Organic: organic dyes, porphyrins, and organic semiconducting polymeric nanoparticles [68,69,70,71];
(iv) Semiconductor: copper selenide (CuSe) and cadmium selenide (CdSe) nanoparticles, and cadmium telluride (CdTe) quantum dots [72,73,74];
(v) Hybrid: core–shell nanoparticles [75,76].

Among various PTAs, organic nanomaterials are often highly biocompatible and biodegradable, exhibiting excellent NIR light absorption compared to many inorganic nanomaterials. Organic dyes can also be conjugated with various biomolecules, including DNA primers, amino acids, nucleotides, and proteins (including antibodies), for use in molecular imaging [77]. The conjugation discussed in this work involves DNA primers rather than genomic DNA; notably, DNA primers are short oligonucleotides that do not carry genetic information and are employed solely as functional probes and marker elements.

Conversely, many inorganic PTAs demonstrate high photothermal efficiency and photostability due to their intrinsic optical properties, including localised surface plasmon resonance (LSPR). Electrons on the surface of these materials vibrate in synchrony when interacting with electromagnetic radiation. Due to this vibration, heat is generated, with the most significant effect occurring at a specific wavelength, known as the LSPR wavelength. Gold-based nanomaterials, such as gold nanoshells, nanorods, nanocages, and nanostars, are among the most analysed examples [78]. However, organic nanomaterials have several limitations, such as poor photothermal stability, low PCE, and complex synthesis.

Despite its advantages over traditional chemotherapy and radiotherapy, PTT alone still faces the risk of tumour recurrence due to its invasive nature [79]. Therefore, a combination of PTT with other treatment methods, including radiotherapy, chemotherapy, and immunotherapy, is gaining popularity. For instance, PTT combined with thermo-chemotherapy enables controlled drug release through light and/or heat stimulation. This dramatically increases the concentration of drugs in the targeted area and addresses the overdosing challenge in healthy tissues [80,81,82]. However, a few drawbacks, such as non-specific PTA distribution, reduce tumour selectivity and pose issues that need to be addressed for clinical translation.

Thus, it has become crucial to advance a theranostic system with therapeutic, imaging, and targeting capabilities. Nanoparticles exhibiting both magnetic properties [83,84,85] and NIR absorption are well-suited to meet these requirements. Their magnetic behaviour enables accumulation at the tumour site under an external magnetic field, while their optical properties allow them to convert NIR irradiation into localised heat for effective PTT. Additionally, these magnetic nanoparticles can serve as contrast agents for MRI, enhancing the precision of both diagnosis and therapy. Among these, iron oxide NPs (Fe_3_O_4_) stand out due to their strong magnetic responsiveness, efficient photothermal conversion, and biocompatibility. The following sections of this review will explore in detail the specific mechanisms and applications of Fe_3_O_4_ NPs in light-based cancer therapies.

## 4. Photodynamic Therapy (PDT)

PDT is a well-established, minimally invasive approach to cancer treatment, and has been employed in clinical settings for over three decades. It relies on three fundamental components:

(i) Light of a specific wavelength;
(ii) A PS that preferentially accumulates in tumour tissues;
(iii) Molecular oxygen present in the target tissue, which is particularly essential for Type II photodynamic reactions.

Among these, the careful selection of light parameters is especially critical. Unlike PTT, wavelengths above 850 nm do not provide enough photon energy to excite photosensitisers to an excited state capable of efficiently generating ROS. Therefore, the optimal wavelength range for PDT lies between 600 and 850 nm, commonly referred to as the “phototherapeutic window” [86,87,88,89]. This range offers optimal tissue penetration while still supplying sufficient energy for ROS production. Wavelengths below 600 nm, although more energetic, are strongly absorbed by tissue and can increase the risk of skin photosensitivity, particularly when the photosensitiser exhibits significant absorption at these wavelengths. Therefore, skin photosensitivity is determined by the combined effect of the light wavelength and the absorption characteristics of the photosensitiser, rather than by the wavelength alone.

Upon light activation, the PS undergoes photochemical reactions that may involve energy or electron transfer processes, leading to the formation of cytotoxic ROS that induces localised damage to cancer cells [7,90].

**Figure 3:**
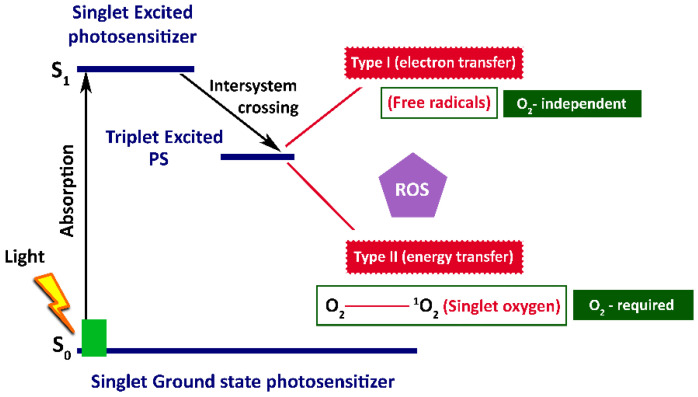
Schematic representation of photosensitiser-mediated ros formation in PDT.

The photodynamic process is initiated through photoirradiation, which excites the PS and triggers photochemical reactions within the tumour cells [91,92,93,94]. Unlike PTT, PDT is characterised not only by singlet ground state (S_0_), and singlet excited states (S_1_, S_2_, S_3_) but also by a longer-lived triplet excited state (T_1_) [95]. Following light absorption, PS molecules are excited to the S_1_ state and then undergo the non-radiative process known as intersystem crossing (ISC) to the T_1_ state (Figure 3).

The fact that PS molecules remain in the triplet excited state for a long time enables them to easily react with nearby substrate molecules like unsaturated lipids, proteins, or nucleic acids [96,97,98,99]. Depending on the reaction pathway, PDT can proceed through Type I and Type II.

(i) Type I: PS molecules in a triplet state directly interact with a biological substrate, generating intermediate free radicals, including highly reactive radicals such as hydroxyl radicals (^•^OH) and superoxide anions (O_2_^•−^). These reactions are not strictly oxygen-dependent. However, in the presence of molecular oxygen, the generated radicals may further react to produce additional ROS, which, in turn, damage membranes of tumour cell organelles, causing their destabilisation and subsequent cell destruction [100,101].
(ii) Type II: PS molecules in a triplet state interact with tissue molecular oxygen and generate highly toxic singlet oxygen (^1^O_2_), which is responsible for the oxidation of tumour cell structures [102].

Both hydroxyl radical (^•^OH) and superoxide anion (O_2_^•−^), formed in Type I, and singlet oxygen (^1^O_2_), formed in Type II, act as ROS in PDT [103].

While Type II reactions (^1^O_2_ production) take place in most of current PDT applications, recent studies indicate that therapeutic efficacy does not always correlate with singlet oxygen quantum yield. Instead, the PS’s ability to undergo Type I reactions may play a more crucial role in tumour destruction, particularly in hypoxic tumour microenvironments where oxygen is limited [104]. However, the precise correlations between ROS generation and tumour ablation mechanisms in PDT remain under investigation.

The clinical relevance of PDT was first demonstrated in 1982 when Hayata et al. [105] administered a flexible endoscope for PDT and documented effective therapy for bronchial cancer. Photoradiation therapy (pRT) was performed on 14 individuals, with 13 diagnosed with lung cancer and one with atypical squamous metaplasia after receiving an intravenous dose of a hematoporphyrin derivative (HpD). The HpD demonstrated selective accumulation in malignant tissues and was subsequently activated using red laser light at a wavelength of 630 nm. Approximately 48 h after the injection of HpD (2.5–4.0 mg/kg), the lesions were exposed to laser irradiation through a fibreoptic bronchoscope. One patient experienced complete tumour regression, remaining disease-free for 16 months. In 12 cases involving advanced lung cancer, localised therapeutic responses were achieved.

Further validation came in 1992 when Kato et al. [106] treated early-stage gastric cancers using either HpD or Photofrin II as PS. These compounds were administered intravenously and subsequently activated using a 630 nm laser source, administered 48 to 72 h after injection. Complete remission was observed in 11 patients, corresponding to 12 lesions, yielding a 60% success rate. These clinical outcomes confirmed the potential of PDT as a curative treatment for early-stage tumours.

Despite being in clinical trials for over three decades, PDT still faces technical challenges. One of the primary difficulties in PDT lies in optimising light distribution to achieve uniform activation of the PS, which is essential for consistent treatment outcomes. Although the use of wavelengths within the phototherapeutic window offers advantages, effectively delivering light remains a significant challenge. This is particularly true in intraoperative settings such as thoracic and cranial PDT, where accessing and uniformly illuminating large or irregular tissue surfaces is complex [107,108]. The success of PDT is influenced not only by the selected wavelength but also by the light source, which should align with the absorption characteristics of the PS (based on excitation and action spectra), as well as the specific disease attributes, including lesion location, size, accessibility, and tissue type. Additionally, several light delivery parameters play a crucial role in determining therapeutic efficacy. These include total fluence (J/cm^2^), fluence rate (W/cm^2^), exposure time, and delivery method (either continuous or fractionated). These variables significantly impact treatment outcomes. Research has shown that lower fluence rates may enhance PDT effectiveness [109,110]. For instance, in a Colon 26 tumour model, light fluence rates ranging from 0 to 224 mW/cm^2^ were tested [111]. It was observed that a high fluence rate of 75 mW/cm^2^ led to rapid oxygen depletion, thereby reducing treatment efficacy, while lower fluence rates were associated with improved tumour control. Furthermore, the fluence rate can alter vascular permeability, which also influences the overall PDT performance.

Light parameters alone, however, do not determine the effectiveness of PDT. PS characteristics, such as limited photostability, poor water solubility, and suboptimal pharmacokinetics, also significantly impact therapy [112]. Many PSs, for instance, Photofrin, although clinically approved for certain cancers, exhibit prolonged skin photosensitivity and lack tumour-specific accumulation [113]. These drawbacks increase the risk of damaging the surrounding healthy tissues.

One of the major challenges in the clinical application of PDT is the insufficient selective accumulation of PS in tumour tissues [114]. For treatment to be efficient, the PS molecules must predominantly concentrate in the disease areas, such as malignant tissues. This tumour-specific accumulation ensures the reduction in toxicity-induced damage to healthy cells within the therapeutic illumination field. Overcoming these barriers requires innovative strategies; among these, nanotechnology-based approaches hold significant promise [115].

To date, a variety of nanoplatforms, including gold nanoclusters [116], carbon-based nanomaterials [117], and quantum dots [118], have been investigated as PS delivery systems for PDT. However, the clinical translation of these conventional nanomaterials remains limited due to issues such as high toxicity and low PS loading capacities, which, in turn, result in subtherapeutic PS concentrations in tumour tissues.

In this context, Fe_3_O_4_ NPs have emerged as a promising multifunctional platform for PDT. They can enhance PS accumulation and selective delivery, as well as improve ROS generation efficiency. The following sections will explore the role of Fe_3_O_4_ NPs in PDT, focusing on their underlying mechanisms and recent advancements in this field.

## 5. Fe_3_O_4_ Nanoparticles in PTT and PDT

As mentioned, both PTT and PDT offer promising potential in cancer treatment. However, each modality is associated with specific limitations. For instance, PTT’s limitations include poor targeting of tumours, uneven heating, and the potential to damage nearby healthy tissues. Likewise, PDT is limited by difficulties in even light delivery, effective PS activation, and a dependence on sufficient oxygen presence for ROS generation.

All these limitations have led to a demand for the development of an integrated theranostic strategy—one that combines diagnostic and therapeutic functions with precise tumour targeting. In this context, Fe_3_O_4_ NPs have emerged as a promising solution due to their structure and physical properties. These NPs not only enable magnetic targeting and MRI contrast enhancement but also facilitate efficient heat generation (PTT) and ROS production (PDT) under light irradiation.

### 5.1. Electronic and Crystal Structure of Fe_3_O_4_

A deeper understanding of the electronic and crystal structure of Fe_3_O_4_ is crucial, as it governs the NP’s optical absorption, light–matter interaction, and photothermal or photodynamic behaviour.

Numerous studies have been conducted on the electrical structures of bulk iron oxides [44,119]. Crystal field theory can be used to describe the crystal structure of Fe_3_O_4_. Fe_3_O_4_ belongs to the spinel group of oxides and it crystallises in a face-centred cubic (fcc) pattern [119]. Its crystal structure contains oxygen anions (O^2−^) forming a close-packed array, with iron cations (Fe^2+^ and Fe^3+^) occupying interstitial tetrahedral (A) and octahedral (B) sites [44,119]. Fe_3_O_4_ exhibits an inverse spinel structure with the following formula:

(5) [Fe^3+^]A[Fe^2+^Fe^3+^]BO_4_

In this configuration, ferric (Fe^3+^) ions occupy both tetrahedral and octahedral sites, while ferrous (Fe^2+^) ions are located only in octahedral positions. The lattice parameter is around 0.84 nm, and each unit cell contains 32 O^2−^ anions and 24 Fe cations, dispersed across 8 tetrahedral and 16 octahedral sites [120,121].

Divalent cations occupy half of the octahedral sites in stoichiometric magnetite, which has a 0.5 Fe^2+^ to Fe^3+^ ratio. Trivalent cations are equally distributed between the tetrahedral sites and the remaining octahedral sites. The band gap of Fe_3_O_4_ is located between the valence band O(2p) and the empty Fe(4s). O(2p) and Fe(4s) have different interbands in between. The octahedral site is characterised by d orbitals that split into t_2g_ (set of three orbitals, lower energy) and e_g_ (a set of two orbitals, higher energy) crystal fields. In comparison, the tetrahedral site is characterised by the splitting of d orbitals into e (a set of two orbitals, lower energy) and t_2_ (a set of three orbitals, higher energy) crystal fields. This splitting creates a series of possible transitions that absorb light energy (Table 1).

Thus, in Fe_3_O_4_, the band gap between the O(2p) and the empty Fe(4s) is between 4 and 6 eV [125]. When photon energy couples with the energy transitions of Fe^2+/3+^ into the Fe_3_O_4_ crystal, light extinction increases. These results indicate that the most absorption occurs at the incident photon energy comparable to the energy gap between O(2p) and (eg) of the octahedral site (Table 1). A substantially smaller amount of absorption may occur due to photon energies that are farther from this threshold (3.1 eV), particularly in the near-infrared spectrum. The predicted energy difference between O(2p) and eg of the octahedral site is thus comparable to the direct band-gap value for Fe_3_O_4_.

These electronic and crystal structure characteristics of Fe_3_O_4_, particularly the distribution of Fe^2+^/Fe^3+^ ions, site-specific d-orbital splitting, and energy band transitions, play a pivotal role in determining its performance in both PTT and PDT.

Selected studies on the application of Fe_3_O_4_ nanoparticles in PTT and PDT combined cancer therapies are discussed in detail below, and recent preclinical trials are summarised in Table 2.

### 5.2. Fe_3_O_4_ NPs in PTT

Fe_3_O_4_ NPs serve dual roles in PTT, functioning as standalone PTAs or as components in hybrid platforms with other PTAs, thereby supporting strong NIR absorption and MRI-guided therapy [138,139,140]. Several factors, including particle size, shape, morphology, surface modification, laser power density, and wavelength, influence their photothermal efficiency under NIR laser irradiation.

Chu et al. [141] reported sufficient photothermal effects of Fe_3_O_4_ NPs, activated by red and NIR laser irradiation. High efficiency was observed in samples treated with 808-nm laser irradiation for 4 days. According to Fu et al. [142], although the absorption intensity of the Fe_3_O_4_ NPs suspensions in the NIR region is not as high as that of visible light, it is still sufficient to achieve superior photothermal performance. The photothermal performance of the particles was evaluated by monitoring the temperature of aqueous solutions with varying concentrations of Fe_3_O_4_ NPs. Various laser power densities and wavelengths were used to irradiate these suspensions. It was found that suspensions with higher Fe_3_O_4_ NPs content exhibited higher temperatures when exposed to an 808 nm laser. Ji and Wang [143] reported high photothermal conversion efficiency of Fe_3_O_4_ NP-loaded ALG hydrogels by demonstrating the death of CT26 cancer cells upon irradiation with an 808 nm laser. Authors revealed that the therapeutic efficacy of Fe_3_O_4_ NP-loaded hydrogels was similar to that of Fe_3_O_4_ NPs alone. However, it was emphasised that Fe_3_O_4_ hydrogels will make a significant contribution to future in vivo and clinical studies due to their ability to maintain high concentrations at injection sites and significantly reduce systemic toxicity when administered intravenously.

The effects of particle size, shape, and surface modification on heating capacity under NIR laser exposure for PTT were studied using iron oxide NPs with average particle sizes ranging from 9 and 78 nm [144]. Despite differences in NP properties, no significant variations were observed in photothermal efficiency. Additionally, octahedral particles of approximately ∼32 nm, coated with dextran, were used for an in vitro study to assess the potential of this material to induce cell death under photothermal conditions. It was concluded that both particle concentration and laser power significantly impacted the reduction in cell viability. Only ten minutes of laser light exposure reduced cell viability to 11% of the cell population under the most extreme conditions of laser power and nanoparticle concentration. Interestingly, the researchers suggested that magnetic nanoparticles, when not efficiently internalised by cells, hold potential for photothermal therapies, specifically in conditions where extracellular particles are effective. This also demonstrates the importance of the rate at which iron nanoparticles accumulate inside and outside cells in terms of the “In&Out” model in preclinical and clinical applications.

In a study by Shen et al. [145], the authors compared individual magnetic Fe_3_O_4_ NPs (15 nm) with Fe_3_O_4_ clusters (225 nm) that shared the same nanocrystalline structure, finding that clustering significantly increased NIR absorption. Following exposure to an 808 nm laser, the viability rates of A549 cells indirectly caused by Fe_3_O_4_ clusters and nanoparticles were 72.8% and 14.5%, respectively. Flow cytometry analysis revealed that the in vitro PTT mechanism was primarily triggered by cell apoptosis rather than necrosis. Given that high Fe_3_O_4_ nanoparticle uptake in the first few days is important for photothermal cancer treatment, especially when repeated NIR irradiation therapy is required, the authors demonstrated that the Fe_3_O_4_ nanoparticles they synthesised could be slowly cleared from tumours after treatment.

An interesting study by Guo et al. [146] described the synthesis of monodisperse Fe_3_O_4_ NPs in four different sizes (60, 120, 200, and 310 nm) with identical surface chemistry. Tumour models were constructed using multicellular tumour spheroids—three-dimensional cell aggregates that mimic primary malignant tumours with micrometastases. Using this model, it was found that particles sized at 60 nm had the best penetration and distribution within tumours, resulting in the highest levels of cell death. Furthermore, larger Fe_3_O_4_ NPs exhibited greater accumulation in tumours, leading to improved tumour growth prevention. Although small nanoparticles can easily leak microvessels and penetrate deep into the tumour matrix, the reason they accumulate less in tumours compared to larger nanoparticles is believed to be their ability to move faster in blood vessels and their higher probability of exiting the tumour. Furthermore, in spite of the fact that NPs larger than 200 nm in diameter appear inadequate for in vivo applications due to their low extravasation capabilities, they can demonstrate significant photothermal ablation efficacy against tumours as photothermal delivery agents when accumulated in large quantities. In this case, destruction of tumour microvessels through photothermal ablation under NIR laser irradiation can disrupt nutrient supply and effectively inhibit tumour growth.

However, despite their advantages, pure Fe_3_O_4_ NPs have several drawbacks. The primary limitation is their low molar absorption coefficient in the NIR region, which results in relatively weak photothermal efficiency. Additionally, their high density of free electrons necessitates significantly higher excitation energies compared to NPs of other metals, complicating their application.

Due to these constraints, Fe_3_O_4_ NPs are typically combined with other PTAs to create hybrid nanocomposite materials. Moreover, strategies including surface functionalisation with various materials enable targeted delivery to tumour cells. For example, Zhou et al. [147] suggested using an NdFeB magnet for the targeted delivery of PEGylated Fe@Fe_3_O_4_ nanoparticles to a grafted HeLa tumour. Upon the employment of this magnet, it was observed that the tumour temperature after irradiation was twice as high as when the magnet was not used.

A pH-sensitive, dual-targeting magnetic nanocarrier for cancer chemo-photothermal synergy, referred to as sample MGO, was developed by incorporating Fe_3_O_4_ NPs into graphene oxide [148]. The nanocarrier was functionalised with polyethylene glycol (PEG) and cetuximab (CET), an epidermal growth factor receptor (EGFR)-targeting antibody, to produce MGO-PEG-CET, designed for the targeted delivery of doxorubicin (DOX). Cytotoxicity tests demonstrated that MGO-PEG-CET/DOX exhibited a lower IC50 value (1.48 µg/mL) compared to the non-targeted MGO-PEG/DOX. Moreover, the addition of NIR photothermal therapy further decreased the IC50 to 1.17 µg/mL.

The combined magnetic behaviour of Fe_3_O_4_ NPs and surface plasmon resonance of gold (Au) makes them useful in biomedical applications. To take advantage of these properties, core-shell gold-coated iron oxide NPs (Au@IONPs) have been prepared and studied as multifunctional agents for MRI contrast and PTT [149]. In terms of MRI sensitivity, samples presented a transverse relaxivity of 95 mM^−1^s^−1^, with a hydrodynamic radius of 33 nm. Cytotoxicity tests on human oral epidermal carcinoma cells demonstrated low toxicity in the absence of laser exposure. In contrast, the Au@IONPs achieved 70% cell death when exposed to a near-infrared laser at 808 nm. One of the important findings of the aforementioned study is that the nanocomplex is relatively safe, even during long-term incubation periods and their penetration into the cells is strongly time-dependent. These properties, when considered together with the nanocomplex’s significant light absorption capacity, make it a promising material for cancer photothermal therapy or thermal ablation of tumours.

The optical properties of chlorin e6 (Ce6) were demonstrated in a study [150] through the conjugation of Ce6 with iron oxide NPs (Fe_3_O_4_-Ce6). Fe_3_O_4_-Ce6 NPs generated heat under laser irradiation at 808 nm, exhibiting a strong photothermal effect, and no notable temperature change was detected after multiple laser cycles. While a small amount of ROS generation was detected via the Fenton reaction in the group treated with Fe_3_O_4_-Ce6 NPs alone, increased ROS formation within cancer cells was confirmed via the photodynamic effect after combination application with 660 nm laser irradiation. The cytotoxicity of Fe_3_O_4_-Ce6 NPs remained low in the absence of laser irradiation, while efficient C6 glioblastoma cell death was induced upon laser exposure. Considering the good fluorescence properties of Fe_3_O_4_-Ce6 NPs, they were investigated as potential therapeutic agents for both PTT and PDT, and are proposed as a fluorescence imaging-guided combination therapy of cancer.

Fe_3_O_4_ NPs show significant promise in PTT, with their photothermal performance influenced by particle design, laser parameters, and surface functionalisation. While standalone NPs exhibit moderate efficiency, combining them with other materials and targeting strategies enhances their therapeutic potential.

### 5.3. Fe_3_O_4_ NPs in PDT

Similar to their role in PTT, Fe_3_O_4_ NPs offer dual functionality in PDT. They act as active ROS-generating compounds, especially in the presence of hydrogen peroxide (H_2_O_2_) and light. These NPs participate in Fenton-like reactions, producing highly reactive ^•^OH radicals, which further amplify oxidative stress in cancer cells. In addition, Fe_3_O_4_ NPs provide the advantage of magnetic targeting, enabling precise delivery of PS to tumour sites, thereby minimising off-target effects and damage to healthy tissues.

The role of Fe_3_O_4_ NPs in ROS-mediated PDT is closely linked to their inherent catalytic behaviour—particularly their peroxidase-like enzymatic activity. Fe_3_O_4_ NPs have been considered the most important peroxidase-mimicking nanozyme [151]. The first paper devoted to this property of Fe_3_O_4_ NPs was published in 2007 [99]. In that study, Fe_3_O_4_ NPs interact with H_2_O_2_, decomposing it into highly reactive ^•^OH or other ROS, mimicking the action of natural peroxidase enzymes. The iron ions (Fe^3+^/Fe^2+^) on the NP’s surface undergo a cycle of electron transfer reactions. These ions alternate between oxidation states, helping to convert H_2_O_2_ into ROS [152,153,154,155]. Compared to other ROS, H_2_O_2_ has a relatively long half-life. It can pass through biological membranes and cause damage to cancer cells, which is a desired effect in PDT via Fe_3_O_4_ [156,157,158,159,160].

Under physiological conditions and influence of the light, Fe_3_O_4_ NPs and H_2_O_2_ participate in photo-Fenton reactions. These reactions lead to the formation of hydroxyl radicals through one-electron reduction of H_2_O_2_ [161]. Additionally, the ferric ions that are products of the first reaction can then be reduced back to ferrous ions by superoxide anions. The combination of these two reactions is termed the iron-catalysed Haber–Weiss reaction [162]:

H_2_O_2_ + Fe^2+^ →HO^•^ + OH^−^ + Fe^3+^

(6) Fe^3+^ + O_2_^•−^ → Fe^2+^ + O

Notably, oxidative stress toxicity is not caused by the Haber–Weiss reaction as a whole but by the Fenton reaction, which is one specific part of it.

Chen et al. [163] developed a nanosystem coated with the Food and Drug Administration (FDA)-approved poly(lactic-co-glycolic acid) (PLGA), which incorporated Fe_3_O_4_ NPs and chlorin E6 (Ce6), merging PDT with ferroptosis—a form of cell death triggered by iron-catalysed lipid peroxidation.

This system is designed to release Fe^2+^/Fe^3+^ ions and Ce6 in the acidic tumour microenvironment. The released Fe ions can react with intracellular H_2_O_2_, initiating the Fenton reaction and leading to the accumulation of ROS and lipid peroxidation. Simultaneously, under laser exposure, Ce6 contributes to additional ROS generation, enhancing the PDT effect and further promoting ferroptosis. Overall, the combined action of PDT and ferroptosis, triggered by the Fe_3_O_4_ NPs, demonstrates a potent antitumour effect.

Similarly, the hybrid nanomaterial Fe_3_O_4_@Cu-TCPP was formed by incorporating polyvinylpyrrolidone (PVP) onto its surface via hydrophobic interactions [164]. The combination of iron oxide NPs with Cu-TCPP enhanced the photo-Fenton process, significantly boosting hydroxyl radical generation under 660 nm laser irradiation, independent of O_2_ levels. In vivo studies demonstrated that Fe_3_O_4_@Cu-TCPP platform enabled T2-weighted MRI and facilitated the combination of CDT and PDT, thereby effectively damaging tumours in vivo.

Beyond catalysing ROS production, Fe_3_O_4_ NPs not only enable the Fenton reaction that amplifies ROS production in cancer cells but also serve as effective carriers for PS. Combining this ROS amplification with targeted delivery mechanisms further enhances overall therapeutic effectiveness, enabling more precise treatment of tumour tissues while minimising damage to healthy cells and improving the therapeutic outcomes of PDT [165,166].

For instance, small-sized (<20 nm) Fe_3_O_4_ NPs embedded within porphyrin-grafted lipid nanoparticles (Fe_3_O_4_@PGL NPs) demonstrated excellent porphyrin loading, low dark toxicity, and significant photodynamic effects against HT-29 cancer cells in vitro. The released Fe ions played a direct role in promoting Fenton reactions, while macrophage-mediated ROS production further increased cytotoxicity [167].

Fe_3_O_4_@Dex-TPP NPs were synthesised via co-precipitation in the presence of triphenylphosphine (TPP)-grafted dextran (Dex-TPP) and Fe^2+^/Fe^3+^ ions, incorporating additional MRI potential [168]. To improve the efficiency of PDT, protoporphyrin (PpIX) and a glutathione-responsive PEG were conjugated to the Fe_3_O_4_@Dex-TPP NPs, resulting in the formation of Fe_3_O_4_@Dex/TPP/PpIX/ss-mPEG. The nanosystem demonstrated mitochondrial targeting and ROS generation through combined photoinduced and Fenton reactions, considerably enhancing PDT efficiency in tumour cells.

A recent investigation [169] introduced a multifunctional Fe_3_O_4_@ZnO NPs-based PS system co-delivered with an anti-EGFR antibody, brusatol, and Nrf2-siRNA, in order to enhance the effectiveness of PDT. Executing a ‘Trojan Horse’ strategy, this platform utilises the anti-EGFR antibody to bypass cellular defences, allowing the nanoparticles to penetrate deep into malignant cells before releasing their therapeutic payload [170]. However, the effectiveness of this targeting mechanism is significantly dependent on the ‘Protein Corona’ phenomenon, characterised by the coating of nanoparticle surfaces with proteins in the biological environment. Since undesirable protein adsorption can mask the functionality of anti-EGFR ligands, surface modification of the Fe_3_O_4_@ZnO system is crucial for both maintaining the accuracy of the ‘Trojan horse’ targeting and evading the immune system [171,172]. Consequently, this advanced nanoplatform overcomes several challenges associated with traditional PDT by leveraging multiple synergistic strategies. The Fe_3_O_4_ core provides magnetic responsiveness, enabling targeted delivery to tumour sites under an external magnetic field. In vitro assessments demonstrated a significant increase in ROS production, by 191.09 ± 10.02% in cutaneous squamous cell carcinoma cells, which corresponded to an 80.43 ± 9.37% decrease in cell viability. Additionally, in vivo tests using nude mice showed a tumour inhibition rate of 76.30 ± 5.12%, indicating strong anti-tumour activity.

Another study [173] introduced a multifunctional nanoplatform based on Fe_3_O_4_ NPs which were surface-engineered to carry the PS chlorin e6 (Ce6) and folic acid (FA), aiming to enhance the performance of PDT. This system facilitated magnetic targeting for precise tumour site localisation and folate receptor-mediated uptake, resulting in a marked increase in singlet oxygen (^1^O_2_) generation under 660 nm laser exposure—a wavelength chosen for its deep tissue penetration.

In one study [174], a novel nanoplatform comprising mesoporous Fe_3_O_4_@TiO_2_ microspheres was developed to enhance NIR-light-triggered CDT, PDT, PTT, and chemotherapy. Titanium dioxide (TiO_2_), a potent PDT agent, was limited by the hypoxic conditions within the tumour microenvironment. To address this, Fe_3_O_4_, a peroxidase-like enzyme, was incorporated into the platform to catalyse the breakdown of H_2_O_2_ in the cytoplasm, generating O_2_ and alleviating tumour hypoxia, which, in turn, promotes ROS production. The cancer-killing potential was further improved by loading the chemotherapeutic agent doxorubicin (DOX), which can be efficiently released upon NIR stimulation and slight acidification. With a high saturation magnetisation of 20 emu/g, the nanoplatform is also well-suited for magnetic targeting.

To enhance the delivery of PS to tumour tissues and improve the efficacy of PDT, Fe_3_O_4_ NPs were incorporated into a self-assembled stomatocyte-like structure made from poly(ethylene glycol)-block-polystyrene (PEG-b-PS), forming nanomotors for the targeted delivery of zinc phthalocyanine (ZnPc) [7]. These hybrid nanomotors are attracted to tumour tissues by the magnetic properties of the Fe_3_O_4_ NPs. Once internalised by cancer cells, the iron oxide NPs catalyse the breakdown of endogenous H_2_O_2_, leading to the generation of O_2_, which acts as a driving force for the nanomotor movement. This movement enhances the distribution of ZnPc, thus expanding the ROS-reactive area and improving PDT efficiency. Additionally, the O_2_ generated supports the PDT process, ensuring optimal performance.

The effective delivery of iron oxide nanoparticles (IONPs) to tumour tissue is primarily based on the principle of increased vascular permeability and poor lymphatic drainage in malignant neoplasms. This phenomenon allows the nanoparticles to selectively accumulate within the tumour, where they remain for an extended period, creating a therapeutic concentration [175]. The rapid and defective angiogenesis in tumours leads to the formation of large fenestrations in the vascular endothelium, ranging from 100 nm to 2 µm [176]. Due to the EPR effect, this “leaky” vascular structure allows for the selective extravasation of IONPs into the interstitial environment. The dysfunctional lymphatic drainage system in the tumour microenvironment prevents particle clearance, allowing them to remain in the extracellular space for an extended period and creating a “therapeutic depot.”

However, modern nanomedicine research emphasises that the EPR effect is not a universal phenomenon and exhibits significant heterogeneity depending on the type of tumour, and the degree of fibrosis [175]. In particular, machine learning-assisted single-vessel analyses have shown that nanoparticle permeability can vary drastically even between different vessels within the same tumour, proving that relying solely on passive diffusion is risky [177]. To compensate for these risks and the instability of the EPR effect, it is necessary to enhance the process with active targeting and physical intervention, rather than relying solely on passive leakage [178]. In particular, exploiting the paramagnetic properties of IONPs (Fe_3_O_4_) by applying an external magnetic field or functionalising their surface with specific ligands allows for overcoming the limitations of passive diffusion by forcing the particles to pass through the tumour’s densest stromal barriers [179,180].

Although a critical size limit of 400 nm for extravasation has been reported in the literature, recent findings demonstrate that IONPs with a diameter smaller than 100 nm are more effective at crossing the dense stromal barrier and can penetrate to the necrotic core of the tumour. Comparative analyses show that passive targeting often leads to the sequestration of large-sized particles (>200 nm) in the perivascular region, which limits the effectiveness of PTT and PDT. Therefore, it is necessary to employ stromal remodelling or active targeting strategies to navigate the complex tumour microenvironment [181,182]. As proposed by Ren et al., combining the EPR effect with a magnetic field or specific ligands (e.g., anti-EGFR) maximises therapeutic precision while minimising systemic toxicity to healthy tissues [169].

Finally, Fe_3_O_4_ NPs boost PDT efficacy via peroxidase-like activity, enabling Fenton-based ROS generation. Their magnetic features permit precise PS delivery, ensuring tumour selectivity. By being incorporated into multifunctional platforms, Fe_3_O_4_ NPs promote synergistic therapeutic effects and imaging, highlighting their promising applications as materials in advanced cancer phototherapy.

## 6. Conclusions

The rapid advancement in nanotechnology has significantly contributed to the development of PTT and PDT. Among the NPs successfully employed in these therapies, Fe_3_O_4_ NPs are recognised as versatile and promising materials for cancer treatment via both photothermal and photodynamic mechanisms. Despite the fact that the Fe_3_O_4_ NPs do not demonstrate remarkable intrinsic optical properties, their magnetic targeting efficiency and catalytic ability to accelerate Fenton reactions position them as indispensable in PTT and PDT applications. Their distinctive electronic properties enhance light absorption and catalysis of ROS generation, which are crucial for efficient tumour destruction.

In PTT, Fe_3_O_4_ NPs have moderate NIR absorption and photothermal conversion efficiency. However, these features can be notably improved through NP size, surface functionalisation, and integration into hybrid nanomaterials. In PDT, the peroxidase-like catalytic activity of Fe_3_O_4_ facilitates the transformation of excess H_2_O_2_ into highly toxic ROS through Fenton and photo-Fenton reactions, enhancing cancer cell destruction. Furthermore, Fe_3_O_4_ NPs function as effective carriers for PS, encouraging targeted delivery alongside selective ROS generation.

The therapeutic efficacy of Fe_3_O_4_ NPs in both PTT and PDT relies on synergistic effects. These effects merge magnetic targeting, photothermal heating, ROS generation, and chemotherapeutic drug delivery within multifunctional platforms. Such synergistic strategies address significant clinical obstacles, including insufficient PS accumulation, tumour hypoxia, and heterogeneous light distribution. However, further studies are required to optimise the design of Fe_3_O_4_ NPs and assess their synergistic capabilities with other nanomaterials. This will maximise therapeutic efficacy and diminish side effects.

Ongoing interdisciplinary efforts integrating materials science, oncology, and nanotechnology are vital. They will facilitate the transition of Fe_3_O_4_-based nanoplatforms from laboratory research to clinical applications. Ultimately, this will simplify the progress towards safe, precise, efficient, and targeted cancer therapies.

## Figures and Tables

**Table 1:** Optical transitions and their energy levels in Fe_3_O_4_ structures [122,123,124].

Transition Type	Energy (eV)
O(2p) → eg (octahedral)	~3.1
t2g → eg (octahedral)	~2.2
e → t2 (tetrahedral)	~0.9
O(2p) → t2 (tetrahedral)	~1.8

**Table 2:** The advantages and key points of recent studies investigating Fe_3_O_4_ NPs based PTT/PDT combined synergistic/dual and trimodal cancer therapies.

Drug Delivery System	Aim/Treatment	Application/Study	Advantages	Reference
Gold-coated Iron Oxide Nanoparticles	Photothermal anti-cancer therapy	in vitro (MCF-7 breast cancer cells)	The effectiveness of magnetic Fe_3_O_4_-Core and Au-Shell with chemically stable, biocompatible, resistant to oxidation, and intrinsic optical properties were examined together within the drug delivery system.	[126]
Doxorubicin-loaded porous Iron Oxide@ Polydopamine (PDA) nanocomposites	MR imaging and synergistic photothermal–chemotherapy of cancer	in vitro	The polymerisation of dopamine provides the huge potential to improve the drug loading capacity, colloidal stability, and photothermal property of Fe_3_O_4_ nanoparticles. The further modification of the nanocomposite surface with PEG allows for prolonged blood circulation lifetime. Furthermore, a multifunctional PION@PDA-PEG nanocomposite combines the functions of magnetic resonance (MR) imaging, PTT, and chemotherapy into one single nanoprobe.	[127]
Iron Oxide nanoparticles coated with Polydopamine (IONs@PDA)	Nano-photothermal agent for treatment of melanoma cancer	in vitro (B16-F10) in vivo (mice)	Nanoparticles initiating photothermal treatment, with a significant apoptosis rate (74%). Photothermal therapy using IONs@PDA proved to be effective in the treatment of melanoma cells (tumour size of <2 mm) without side effects.	[128]
Fe_3_O_4_ nanoparticle clusters	Photothermal ablation of murine melanomas	in vitro (A375 cells) in vivo (BALB/c mice)	Since a very high dosage is needed to generate sufficient hyperthermia by NIR irradiation, lingering magnetite may impose systemic toxicity. Thus, single Fe_3_O_4_ nanoparticles need to be modified to diminish the dosage while maintaining their therapeutic efficacy. Herein, clustered magnetic Fe_3_O_4_ nanoparticles induce a redshift in the light absorption spectra, which enhances light absorbance within the NIR region and improves their utilisation as photosensitisers during PTT to ablate tumours.	[129]
Liposomal Iron Oxide Nanoparticles Loaded with Doxorubicin	Combined chemo-photothermal cancer therapy	in vitro (B16F10 murine melanoma cell line) in vivo (mice)	Since iron nanoparticles have low water solubility, liposomal coating makes them more biocompatible and biodegradable. Moreover, due to its composition of a water/buffer-filled core and an amphiphilic double-layered membrane, both hydrophilic and hydrophobic drugs could be loaded into the liposome.	[130]
Porous hollow copper iron oxide nanoparticles (PHCuFeNPs)	Trimodal chemodynamic-photothermal-chemo anti-tumour therapy	in vitro (4T1 and L02 cells) in vivo (female Balb/c mice)	The pores of PHCuFeNPs enable controlled Cu and Fe ion release, which could drive the Fenton reaction to accomplish chemodynamic therapy (CDT) and accelerate the cisplatin release to achieve chemotherapy. Besides, the immunogenic cell death induced by PHCuFeNPs could activate the T cell-mediated immune system and achieve complete tumour elimination by combining with multi-antitumour therapy.	[131]
Fe_3_O_4_@PDA-PEG-cRGDAA@ Gox (GOx@FeNPs)	Synergistic colorectal cancer therapy	in vitro (CT26 cells) in vivo (Balb/c mice subcutaneous CRC models)	Dual-targeted nano delivery system (GOx@FeNPs) combined with immune checkpoint blocker inhibits colorectal cancer progression by mediating PTT, ferroptosis, and anti-tumour immune response. The tumour targeting ability of Fe_3_O_4_@PDA nanoparticles was enhanced by using the cyclic arginine glycyl aspartate (cRGD) peptide and anisamide (AA). By introducing glucose oxidase (GOx), the Fe^2+^-mediated Fenton reaction was further accelerated, which enhanced cellular ferroptosis.	[132]
Cluster/shell citrate-Fe_3_O_4_/chitosan nanoparticles	Combined magnetic and photothermal therapy	in vitro (BV2 microglia cells)	The first study investigated magnetic clustering using a chitosan polymeric coating where it allows magnetic NPs elongate blood circulation half-time and stabilisation. The design of cluster/shell nanostructures offers several advantages, such as targeted delivery, controlled drug release, higher colloidal stability, and biocompatibility. Moreover, clusters or aggregates of magnetic NPs display significantly enhanced magnetic properties compared to monodisperse NPs.	[133]
Magnetically guided Bi_2_S_3_@C/Fe_3_O_4_ nanoparticles	Enhanced photothermal-radiation synergy in glioblastoma treatment	in vitro (U87MG-Luc and U251 cells) in vivo (female Balb/c mice)	The Bi_2_S_3_ core enables efficient NIR photothermal conversion and X-ray attenuation. The carbon shell enhances colloidal stability and biocompatibility, while Fe_3_O_4_ doping enables magnetic targeting. Using 3D printing, a customised magnetic helmet for mice was produced to establish localised magnetic fields, guiding preferential accumulation of the nanoparticles at intracranial tumour sites.	[134]
Multifunctional nanoplatform based on Fe_3_O_4_@Au nanocomposites (NCs)	Single NIR light-triggered PTT/PDT synergistic therapy of cancer	in vitro (cervical cancer (HeLa) cells)	Due to the superparamagnetic nature of Fe_3_O_4_ cores and the presence of Au shells, these NCs could permeate into and accumulate in tumour regions via enhanced permeability and retention (EPR) effect. Furthermore, Fe_3_O_4_ NPs endowed Fe_3_O_4_@Au NCs with targeting ability, which would further promote the distribution of nanotherapeutic agents in cancer cells with an external magnetic field, thus reducing damage of PTT/PDT to healthy cells. This even leads to tumour ablation at mild temperatures, realising PTT/PDT dual-modal therapy of tumours, making Fe_3_O_4_@Au NCs promising multifunctional nanoplatforms for cancer therapy.	[135]
Aluminium phthalocyanine tetra sulfonate (AlPcS_4_) conjugated glutamine-coated iron oxide nanoparticles (IONs)	Photodynamic therapy of Ehrlich tumour-bearing mice	in vivo (albino mice)	AlPcS_4_ exhibits high absorption in the red-light region around 670 nm, enabling deeper tissue penetration. It possesses a high singlet oxygen quantum yield and can emit fluorescence at 635 nm. Using L-glutamine as a coating agent for the IONs and the conjugation of AlPcS_4_ offered advantages in terms of stability and biocompatibility. Moreover, L-glutamine-coated IONs can exhibit good water-dispersibility and optimal magnetic moment, which are essential for multimodal cancer treatment.	[136]
Methylene blue-encapsulated superparamagnetic iron oxide nanoparticles coupled with NIR-responsive upconversion carbon dots	Improved photodynamic therapy	in vitro (breast (MCF-7), colorectal (HCT-116), pancreatic (PC3), liver (HepG-2), epithelial (MCF-12F), and fibroblastic (BJ-1) cell lines)	As a low-toxic photosensitiser methylene blue (MB) can produce singlet oxygen. Upconversion carbon dots (CDs) enable a well-defined nanoscale interface for energy transfer. PEG functionalised on the surface of superparamagnetic iron oxide (SPIONS) allows for the formation of PEGylated superparamagnetic iron oxide nanoparticles (PEG-SPIONS), which can circulate in the bloodstream for longer periods by avoiding detection and clearance by immune cells.	[137]

## Data Availability

No new data were created or analysed in this study. Data sharing is not applicable to this article.

## Abbreviations

The following abbreviations are used in this manuscript:

BLL	Beer–Lambert Law
CDT	chemodynamic therapy
Ce6	chlorin e6
CET	cetuximab
EGFR	epidermal growth factor receptor
EPR	enhanced permeability and retention
FA	folic acid
fcc	face-cantered cubic
FDA	Food and Drug Administration
Fe_3_O_4_ NPs	iron oxide nanoparticles
HpD	hematoporphyrin derivative
ILP	interstitial laser photocoagulation
IONP	Iron oxide nanoparticles
ISC	intersystem crossing
LA	laser ablation
LITT	laser interstitial thermal therapy
LSPR	localised surface plasmon resonance
MRI	Magnetic Resonance Imaging
NIR	near-infrared
NPs	nanoparticles
PCE	photothermal conversion efficiency
PDT	photodynamic therapy
PEG	polyethylene glycol
PLGA	poly(lactic-co-glycolic acid)
PpIX	protoporphyrin
pRT	photoradiation therapy
PS	photosensitisers
PTA	photothermal agents
PTT	photothermal therapy
PVP	polyvinylpyrrolidone
ROS	reactive oxygen species
TPP	triphenylphosphine
WHO	World Health Organisation
